# The relationship between xerostomia and oral health-related quality of life in females with polycystic ovary syndrome

**DOI:** 10.1186/s12905-026-04688-5

**Published:** 2026-07-17

**Authors:** Kübra Karaçam, Rahime Zeynep Erdem, Özlem Gül

**Affiliations:** 1 Department of Periodontology, Faculty of Dentistry, Afyonkarahisar Health Sciences University, Afyonkarahisar, 03030 Turkey; 2 Department of Restorative Dentistry, Faculty of Dentistry, Afyonkarahisar Health Sciences University, Afyonkarahisar, 03030 Turkey; 3 Afyonkarahisar State Hospital, Afyonkarahisar, 03000 Turkey

**Keywords:** Polycystic ovary syndrome, Oral health, Xerostomia

## Abstract

**Background:**

Polycystic Ovary Syndrome (PCOS) is a disorder commonly seen in females of reproductive age. Significant associations have been reported between oral inflammation, periodontal diseases, and PCOS. An increase in inflammatory markers may be a factor for quality of life in patients with PCOS.

**Objectives:**

The aim of this study was to evaluate the relationship between xerostomia and oral health-related quality of life in females with PCOS.

**Methods:**

In face-to-face interviews, patients diagnosed with PCOS and a healthy control group completed a questionnaire including demographic data, the Oral Health Impact Profile (OHIP-14) and the Xerostomia Inventory. The data obtained were analyzed statistically in the IBM SPSS vn. 27 program.

**Results:**

The OHIP-14 total points and the xerostomia points were determined to be statistically significantly higher in the PCOS group than in the control group (*p* < 0.05). A positive, moderate-level correlation was seen between the OHIP-14 total points and the xerostomia points (*p* < 0.05).

**Conclusion:**

The study results showed that xerostomia was associated with PCOS and the oral health-related quality of life was affected in these patients. In females diagnosed with PCOS, keeping oral health under control can improve quality of life. It highlights the importance of integrating routine oral health assessments into the standard clinical management of women with PCOS. Healthcare professionals should adopt a multidisciplinary approach to identify and manage oral health problems that could adversely affect patients’ quality of life.

## Introduction

 Polycystic ovary syndrome (PCOS) is a metabolic and hormonal disorder affecting 4%-21% of women worldwide, whose aetiology remains incompletely understood [1, 2]. hyperandrogenaemia is one of the important pathological characteristics of PCOS and can be seen together with chronic anovulation [3]. Females with PCOS are at increased risk of hormonal and metabolic dysfunction, the latter affecting approximately two-thirds of these patients [4, 5]. These patients are at greater risk of developing insulin resistance, obesity, dyslipidaemia, cardiovascular disease, and endometrial carcinoma [3]. PCOS has also been associated with psychological effects such as depression, anxiety, and diminished quality of life. Previous studies have reported that PCOS is associated with alterations in oral health, including quantitative and qualitative changes in saliva [6, 7]. Chronic disorders such as PCOS can significantly affect quality of life, warranting careful evaluation.

Oral health-related quality of life (OHRQoL) is a multi-dimensional concept that is used to define the physical, emotional, and social aspects of certain diseases or treatments [8]. As oral health is an inseparable part of general health, any disease affecting the mouth affects quality of life [9, 10]. Thus, OHRQoL reflects the impact of oral health on an individual’s quality of life [11], and is assessed using patient-centred approaches. When OHRQoL scores are combined with clinical criteria, they provide an appropriate framework for evaluating treatment needs and outcomes [12]. The Oral Health Impact Profile-14 (OHIP-14) is one of the most widely used instruments for this purpose.

Xerostomia is the subjective sensation of dry mouth, which may arise from numerous local or systemic factors, including direct damage to salivary gland tissue [13]. Although numerous studies have investigated salivary parameters in patients with PCOS, studies directly evaluating xerostomia symptoms remain limited. Most research has primarily focused on periodontal health, salivary biomarkers, and alterations in the oral microbiome in the PCOS population [14–16]. Previous studies have demonstrated salivary alterations, including changes in pH and increased levels of inflammatory markers [17, 18]. These findings suggest possible but not yet fully established mechanisms linking PCOS to oral health complications. Inflammatory cytokines have been reported to impair salivary gland function in other conditions, and by analogy, may contribute to reduced fluid secretion by affecting acinar cells and epithelial integrity, as demonstrated particularly in studies on Sjögren’s syndrome [19]. However, this association should be interpreted with caution, as whether the same mechanism operates in PCOS remains to be confirmed. In this context, the salivary alterations observed in PCOS could hypothetically contribute to the development of xerostomia, although direct evidence for this pathway is currently lacking. Among the few studies that have assessed xerostomia in patients with PCOS, Surmelioğlu et al. [17] reported no significant difference in salivary flow rate in this population, but observed an increase in salivary viscosity. Increased salivary viscosity may be associated with the development of xerostomia symptoms.

Furthermore, salivary testosterone concentrations have been reported to be significantly higher in women with PCOS [14, 20]. Given that sex steroid hormones are known to influence salivary gland function in other contexts, it has been hypothesised that elevated salivary testosterone in PCOS could contribute to the development of xerostomia, although direct evidence for this pathway in PCOS is currently lacking [21]. 

Xerostomia is a multifactorial condition that can significantly affect OHRQoL [22]. Therefore, a better understanding of the potential relationship between PCOS and xerostomia is important for both the prevention of oral health complications and the improvement of quality of life.

Evaluation of the relationship between xerostomia and OHRQoL in PCOS patients can help clinicians to prioritise the planned treatment. The aim of this study was to investigate OHRQoL and its relationship with xerostomia in patients with PCOS.

## Material and method

Approval for this survey study was granted by the Afyonkarahisar Health Science University Non-Interventional Scientific Research Ethics Committee(decision no: 2024\260). All the study participants provided written informed consent. All the study procedures were in compliance with the principles of the Helsinki Declaration.

With reference to a study by Yildirim et al. [23], assuming 0.5 effect range with α = 0.05 (significance level) and 1-β = 0.8 (power), the sample size required was found to be a total of 128 subjects with 64 in each group. A total of 100 females diagnosed with PCOS as the test group and 100 healthy females with regular menstrual cycles as the control group were included in the study. The inclusion criteria for the test group were defined as age between 20 and 40 years, and a diagnosis of PCOS made at least one year previously.

The diagnosis of PCOS was made according to the presence of at least two of the Rotterdam criteria [24] as follows: (1) polycystic ovaries observed on ultrasound scan (the presence of ≥ 12 follicles on one or both ovaries and/or increased ovarian volume, > 10 ml), (2) clinical symptoms of hyperandrogenism (hirsutism score > 7 or hirsutism score based on evident acne) and/or elevated plasma testosterone (> 2.0 nmol/l), (3) interval between menstruations of > 35 days and/or amenorrhea, defined as no vaginal bleeding for at least 6 months. The exclusion criteria for both groups were defined as pregnancy, menopause, the presence of any systemic disease affecting the salivary glands, type 1 or type 2 diabetes mellitus, cardiovascular disease, malignancy osteoporosis, thyroid dysfunction, a psychiatric diagnosis, or the use of antidepressants or allergy medications. Among the participants who met the inclusion and exclusion criteria, women who were diagnosed with PCOS according to the Rotterdam criteria between November 2024 and September 2025 were consecutively recruited into the study group. The control group consisted of individuals without a diagnosis of PCOS who met the same inclusion and exclusion criteria and were also enrolled consecutively. Baseline demographic and clinical characteristics, including age, BMI and educational status, were recorded for all participants. Disease duration (years) was recorded for individuals in the PCOS group.

Then in face-to-face interviews, all the participants completed the Xerostomia Inventory (XI) and the Oral Health Impact Profile (OHIP-14) questionnaires. The OHIP-14 questionnaire version shortened by Slade and Spencer [25] was used to determine the effect of PCOS on OHRQoL. This questionnaire consists of 14 items from seven areas in six subdimensions of functional limitation, pain, psychological discomfort, physical disability, psychological disability, and social disability. Negative experiences of the participants are scored between 0 and 4 points on the scale, giving a total score in the range of 0–56 points, with a higher score indicating a lower OHRQoL. Any score > 14 points is accepted as a sign of low OHRQoL [25]. Translation of the scale into Turkish and validity and reliability studies were confirmed by Mumcu et al. [26]

The Xerostomia Inventory (XI) was used to evaluate the severity of dryness during daily living activities. The XI consists of 11 items assessing dryness of the mouth, eyes, nose, and skin. One item, the standard question “How often does your mouth feel dry?” is scored on a 4-point Likert scale (1: never to 4: always), while the remaining 10 items are scored on a 5-point scale (1: never to 5: very often). The Turkish version of the XI, translated and validated by Sarı et al. [27], was used in this study. The total score is the sum of all item scores, with higher scores indicating more severe xerostomia. This mixed response format reflects the original design of the XI and has been preserved in its validated Turkish version. The total score is the sum of all item scores, with higher scores indicating more severe xerostomia.

### Statistical analysis

Data obtained in the study were analyzed statistically using IBM SPSS vn. 27 software. Descriptive statistics of the data were stated as mean ± standard deviation (SD) or median and interquartile range (25%-75% quartiles) values for continuous variables and as number (n) and percentage (%) for categorical variables. The reliability of the scales used in the study was tested. Conformity of the data to normal distribution was assessed with the Shapiro Wilk test. In the comparisons of two independent groups of data not showing normal distribution, the Mann Whitney U-test was applied. Spearman correlation analysis was applied in the examination of relationships between continuous measurements not normally distributed. When testing the relationships between categorical variables, Fisher’s Exact test was used when the sample size assumption was not met (expected value > 5). In the modelling of a categorical dependent variable with independent variables, Binary Logistic Regression analysis with the Forward Wald approach was used. A value of *p* < 0.05 was accepted as statistically significant. A binary logistic regression model was applied using the Forward Wald method to evaluate the effects of the study variables on group status (PCOS vs. control). The control group was defined as the reference category. Group status was entered as the dependent variable, with age, education level, disability score, and Xerostomia Inventory (XI) score included as independent variables. Variables that were not statistically significant were excluded from the final model according to the Forward Wald selection procedure.

## Results

Reliability analyses were performed to test the consistency and reliability of the responses given by the participants to the questions in the scales. In the PCOS group, the Cronbach alpha reliability coefficient for the OHIP-14 was calculated as 0.820, showing a high level of reliability, and for the XI, the Cronbach alpha reliability coefficient was calculated as 0.879 with a high level of reliability. In the control group, the Cronbach alpha reliability coefficient for the OHIP-14 was calculated as 0.780, showing good reliability, and for the XI, the Cronbach alpha reliability coefficient was calculated as 0.829 with a high level of reliability.

The age and body mass index (BMI) measurements of the PCOS group were determined to be statistically significantly higher than those of the control group (*p* < 0.05) (Table 1).

**Table 1:** Distribution and comparison of age and BMI values according to study groups

	PCOS		Control		Intergroup	
	Mean.±SD.	M(%25-%75IQR)	Mean ± SD.	M(%25-%75IQR)	Test Statistics	*p*
Age(years)	27,94 ± 6,64	27(22–33)	23,42 ± 4,68	23(21–24)	-4,809	< 0,001*
BMI	25,75 ± 4,49	25,3(22,62 − 29,09)	23,13 ± 4,02	22,04(20,59 − 24,87)	-4,509	< 0,001*
Disease duration(year)	5,34 ± 7,86	3(2–6)	-	-		

*p < 0,05

Statistically significant differences were determined between the groups in respect of the OHIP-14 total and functional limitation, physical disability, psychological disability, social disability, and handicap points and the XI points, with higher points obtained in the PCOS group than in the control group (*p* < 0.05). No significant difference was seen between the groups in respect of pain and psychological discomfort points (*p* > 0.05) (Table 2). A statistically significant relationship was found between the education level and the groups (*p* < 0.05).

**Table 2:** Distribution and comparison of OHIP-14 and Xerostomia Inventory scores according to study groups

	PCOS		Control		Intergroup	
	M.±SD	M.(%25-%75IQR)	M.±SD	M.(%25-%75IQR)	Test Statistics	*p*
Functional limitation	1,15 ± 1,31	1(0–2)	0,52 ± 0,85	0(0–1)	-3,818	< 0,001*
Physical pain	2,26 ± 1,79	2(1–3)	1,78 ± 1,62	1(0–3)	-1,903	0,057
Psychological discomfort	3,09 ± 1,76	3(2–4)	3,47 ± 1,70	3,5(2–5)	-1,497	0,134
Physical disability	1,80 ± 1,47	2(1–3)	0,94 ± 1,29	0(0–2)	-4,593	< 0,001*
Psychological disability	1,62 ± 1,68	1(0–3)	0,98 ± 1,23	1(0–2)	-2,554	0,011*
Social disability	1,50 ± 1,40	1(0–2,5)	0,72 ± 1,21	0(0–1)	-4,636	< 0,001*
Handicap	1,42 ± 1,52	1(0–2)	0,52 ± 0,86	0(0–1)	-4,780	< 0,001*
OHIP-14 total score	24,26 ± 13,7	20(12,5–36)	17,33 ± 10,74	14(8–25,5)	-3,620	< 0,001*
Xerestomia Inventory	29,25 ± 8,14	28(23,5–32,5)	25,34 ± 6,41	24,5(20–30)	-3,342	0,001*

*p < 0,05

In the PCOS group, relationships between the demographic characteristics and the OHIP-14 and XI points were examined. A statistically significant, positive, moderate-level correlation was determined between pain and time since diagnosis, between psychological discomfort and time since diagnosis and age, and between social disability and time since diagnosis (*p* < 0.05 for all). A statistically significant, positive, low-level correlation was determined between functional limitation and time since diagnosis, between pain and education level, and between handicap and time since diagnosis (*p* < 0.05 for all). A statistically significant, positive, moderate-level correlation was determined between the OHIP-14 total points and the XI total points and the time since diagnosis (*p* < 0.05) (Table 3).

**Table 3:** Relationships between demographic characteristics and OHIP-14 and Xerostomia inventory scores for the PCOS group

		Disease duration(Year)	Age(Year)	Educational status	BMI
Functional limitation	*r*	0,206	0,145	-0,103	0,136
*p*	0,040*	0,150	0,306	0,177	
Physical pain	r	0,401	0,123	0,205	-0,044
	p	< 0,001*	0,221	0,040*	0,664
Psychological discomfort	r	0,382	-0,010	0,245	-0,104
	p	< 0,001*	0,922	0,014*	0,302
Physical disability	r	0,160	0,016	0,014	0,035
	p	0,112	0,871	0,891	0,732
Psychological disability	r	0,269	0,212	-0,056	0,146
	p	0,007*	0,035*	0,579	0,147
Social disability	r	0,360	0,044	0,042	-0,018
	p	< 0,001*	0,661	0,679	0,858
Handicap	r	0,235	0,175	-0,006	0,161
	p	0,018*	0,082	0,951	0,109
OHIP-14 total score	r	0,454	0,137	0,097	0,031
	p	< 0,001*	0,175	0,336	0,760
Xerestomia Inventory	r	0,406	-0,054	0,196	-0,166
	p	< 0,001*	0,594	0,051	0,099

*p < 0,05 ve r: Correlation coefficient

In the control group, relationships between the demographic characteristics and the OHIP-14 and XI points were examined. A statistically significant, positive, weak-level correlation was determined between psychological discomfort and education level, psychological disability and education level, and between the OHIP-14 total points and education level (*p* < 0.05 for all) (Table 4).

**Table 4:** Relationships between demographic characteristics and OHIP-14 and Xerostomia inventory scores for the control group

		Age(Year)	Educational status	BMI
Functional limitation	*r*	-0,142	0,118	-0,055
*p*	0,160	0,242	0,586	
Physical pain	r	-0,027	0,185	0,110
	p	0,788	0,066	0,275
Psychological discomfort	r	0,123	0,261	0,131
	p	0,224	0,009*	0,195
Physical disability	r	0,078	0,129	0,114
	p	0,438	0,201	0,260
Psychological disability	r	-0,119	0,215	0,085
	p	0,239	0,032*	0,403
Social disability	r	-0,111	0,030	0,076
	p	0,273	0,764	0,451
Handicap	r	-0,178	-0,001	0,080
	p	0,078	0,992	0,433
OHIP-14 total score	r	-0,055	0,205	0,114
	p	0,590	0,040*	0,260
Xerestomia Inventory	r	0,034	0,179	0,041
	p	0,736	0,075	0,688

*p < 0,05 ve r: Correlation coefficient

In the PCOS group, statistically significant, positive, weak-level correlations were determined between the XI points and functional limitation, pain, psychological discomfort, physical disability, and social disability subdimension points (*p* < 0.05 for all). A statistically significant, positive, moderate-level correlation was determined between the XI points and the OHIP-14 total points (*p* < 0.05) (Table 5). In the control group, statistically significant, positive, weak-level correlations were determined between the XI points and the physical disability and social disability subdimension points (*p* < 0.05) (Table 5).

**Table 5:** Relationships between OHIP-14 and Xerostomia inventory scores in groups

		Xerestomia Inventory	
		PCOS	Control
Functional limitation	r	0,230	0,141
	p	0,022*	0,163
Physical pain	r	0,263	0,063
	p	0,008*	0,534
Psychological discomfort	r	0,293	0,133
	p	0,003*	0,187
Physical disability	r	0,212	0,222
	p	0,034*	0,026*
Psychological disability	r	0,097	0,148
	p	0,339	0,143
Social disability	r	0,241	0,264
	p	0,016*	0,008*
Handicap	r	0,118	0,165
	p	0,243	0,103
OHIP-14 total score	r	0,337	0,215
	p	0,001*	0,032

*p < 0,05 ve r: Correlation coefficient

Binary logistic regression analysis was performed to evaluate factors associated with group status (PCOS vs. control). In binary logistic regression analysis, the Forward-Wald selection procedure was used to ensure that only significant variables remained in the model and the final model could be obtained. Using the Forward Wald selection procedure, age, education level, disability scores, and Xerostomia Inventory (XI) scores were retained in the final model and were significantly associated with group status (Table 6). Variables that were not statistically significant, including BMI, were not retained in the final model, as per the Forward Wald variable selection procedure. The model correctly classified 77.4% of the cases. The odds ratio for the XI score was 1.101. Accordingly, each one-point increase in the XI score was associated with a 1.10-fold increase in the likelihood of being in the PCOS group compared to the control group.

**Table 6:** Binary logistic regression analysis for groups

							%95 Confidence interval	
Variables	β	S.H.	Wald	*p*	O.O.		Lower limit	Upper limit
Age(years)	0,106	0,037	8,433	0,004*	1,112		1,035	1,195
E.S.Primary school			21,974	0,001*				
E.S.Middle school	18,873	40192,969	0,000	1,000	157134776,875		0,000	
E.S.High school	0,634	0,850	0,557	0,456	1,886		0,356	9,976
E.S.University	-1,568	0,772	4,123	0,042*	0,208		0,046	0,947
E.S.Vocational school	-1,645	0,901	3,330	0,068	0,193		0,033	1,129
E.S.Doctorate	-1,741	1,643	1,123	0,289	0,175		0,007	4,391
Handicup	0,517	0,175	8,696	0,003*	1,677		1,189	2,364
Xerestomia Inventory	0,096	0,029	11,238	0,001*	1,101		1,041	1,164
Intercept	-4,737	1,466	10,441	0,001*	0,009			
E.S.: Educational status Hosmer - Lemeshow; X^2^= 4,737 ve *p* = 0,785								
Cox and Snell R^2^= 0,348 ve Nagelkerke R^2^= 0,463								

Accuracy rate = 0,774

* p < 0.05,* S.E. *Standard Error,* O.R. *Odds Ratio,* Ref. *Reference

## Discussion

Although studies in the literature have identified oral symptoms in patients with PCOS and evaluated health-related quality of life (HRQoL), to the best of our knowledge no study has specifically evaluated OHRQoL in this population. Therefore, this is the first study to have evaluated xerostomia and OHRQoL in females with PCOS.

Oral symptoms may be associated with many systemic, bacterial, viral, and genetic diseases. It has been suggested in the literature that hormonal changes occurring in PCOS, together with gingivitis and the amount of potential periodontal micro-organisms in the saliva, may hypothetically be associated with alterations in the immune responses of the body, although the precise mechanisms remain to be directly investigated. A higher prevalence of periodontal disease has been observed in patients with PCOS than in systemically healthy individuals and this has been shown to be associated with systemic inflammation [28]. Özçaka et al. reported that PCOS could be associated with gingivitis [29]. Porwal et al. observed that females with PCOS had a higher rate of gingivitis and periodontal destruction compared with those without the syndrome [30]. As the hormonal and inflammatory pathways linking PCOS to xerostomia were not directly investigated in the present study, any mechanistic interpretations should be regarded as hypothetical and based on indirect evidence from the existing literature.

There are also studies reporting that, in addition to periodontal diseases, dental caries occur more frequently in PCOS patients than in healthy individuals. In a study by Hilaloğlu et al., higher DMFT (decayed, missing, filled teeth) index values and rates of *S.mutans* were detected in the saliva of PCOS patients [17, 31]. In addition to studies related to the association between PCOS and oral health, there are also studies that have shown that HRQoL is diminished and that demographic data affect PCOS incidence. Merkin et al. reported a higher incidence of PCOS in females of low socioeconomic status [32]. Consistent with that finding, the results of the current study showed a statistically significant correlation of education level with the PCOS and control groups. It was also seen that lower education level in females with PCOS was associated with higher pain and psychological discomfort scores.

The current study findings showed that time since PCOS diagnosis was associated with an increase in all but the physical disability subdimension scores, and a significant increase in the OHIP-14 total scores and the xerostomia severity. These results are consistent with those of a study by Rzonca et al., which showed that quality of life decreased as the time since diagnosis increased in females with PCOS [33]. 

In the studies in the literature that have evaluated HRQoL in PCOS patients, the SF-36 and PCOSQ have been most commonly used [34]. The PCOSQ is a validated instrument designed specifically to assess HRQoL in PCOS [35, 36], and includes areas of emotions, hirsutism, weight, fertility, and menstrual disorders. Studies that have used the PCOSQ have shown that females with PCOS have functional disorders in some of the measured areas [37]. Other studies using the PCOSQ have commonly reported that excess body weight is a significant source of anxiety for females with PCOS [38, 39]. Two studies reported a strong relationship between weight gain and low quality of life [40, 41]. In the current study, that the BMI of the PCOS group was significantly higher than that of the control group was consistent with the results of the above-mentioned studies.

The SF-36 is the most accepted and one of the most frequently used instruments for the measurement of quality of life. The scale consists of 8 subscales of physical function, physical role, physical pain, general health, vitality, social function, emotional role, and mental health [42]. Previous studies have shown that according to the SF-36, women are observed to have both psychological and physical outcomes associated with PCOS [43–45]. In the current study, although no difference was seen in the psychological discomfort subdimension between the PCOS group and control group, a significant difference was observed in the physical disability and psychological disability values. Jones et al. [36] showed that emotional role had the greatest negative association with HRQoL, while Coffey et al. [46] and Bazarganipour et al. [47] stated that psychological areas were most impacted, as reflected in lower scores in these domains.

Psychological stress has been suggested to be a factor that may be associated with xerostomia and reduced flow rate of saliva [48, 49]. In a study of adults in India, it was reported that OHRQoL, stress, depression and anxiety were associated with xerostomia and unstimulated salivary flow rate [50]. The current study results were consistent with those of a study by Hilaloğlu et al., which stated that salivary viscosity was increased in females with PCOS [31]. Various studies have shown that OHRQoL is significantly associated with xerostomia [51, 52]. The current study results found a significant correlation between the OHIP-14 total scores and xerostomia severity, suggesting that xerostomia may be associated with lower OHRQoL in females with PCOS. However, this association should be interpreted with caution, as the PCOS and control groups differed significantly in age and BMI, and residual confounding from these variables cannot be fully excluded.

The current study participants in the PCOS group were older and had higher BMI values than those in the control group. This was not an unexpected finding, as increased BMI is commonly observed in individuals with PCOS. As the study participants were recruited from patients presenting at the clinic, these differences most likely reflect the natural characteristics of this population. However, these baseline differences between groups represent important limitations that should be considered when interpreting the findings.

Both age and BMI are known to influence oral health and salivary function. Age is associated with reduced salivary flow and increased xerostomia prevalence, and was therefore included as a covariate in the regression analysis. After adjustment for age, the association between xerostomia severity and PCOS remained significant, suggesting that age alone does not fully account for the observed association between xerostomia severity and PCOS. Nevertheless, it should be acknowledged that controlling for age may not have eliminated all age-related confounding, given the magnitude of the age difference between groups.

BMI is also recognised as a factor that may influence salivary gland function and oral health outcomes. Although BMI differed significantly between the groups, it was not retained in the final regression model. This may be because BMI and age are closely related variables individuals with higher BMI tend to also be older and when both are included in the same model, the statistical contribution of BMI may be obscured by its overlap with age. Therefore, the absence of BMI from the final model should not be interpreted as evidence that BMI has no effect on the observed associations. Its potential confounding effect cannot be fully excluded, and this represents an important limitation of the current study. Accordingly, residual confounding due to both age and BMI cannot be fully excluded. Statistical adjustment for a single covariate does not fully replicate the effect of simultaneous multivariate control, particularly when confounders are intercorrelated as is the case with age and BMI in the present study. Therefore, the strength of the observed associations between PCOS, xerostomia, and OHRQoL should be interpreted with caution.

It should also be noted that periodontal status and dental caries were not assessed in the present study. Periodontal inflammation may independently impair salivary gland function and contribute to xerostomia symptoms. It cannot be excluded that unmeasured periodontal differences and dental caries between groups may have contributed to the observed differences in xerostomia severity and OHRQoL scores. The absence of these clinical assessments therefore represents an important limitation. As a result, we cannot determine whether the higher xerostomia severity and lower OHRQoL observed in the PCOS group reflect a direct effect of PCOS itself, an indirect effect mediated by undiagnosed periodontal disease or caries, or a combination of both. As periodontal and dental clinical data were not collected as part of the study protocol, it was not possible to include these variables in the regression model or to conduct sensitivity analyses examining their potential confounding or mediating role. This reflects a limitation in data availability rather than an analytical decision. Future studies should incorporate detailed clinical periodontal and caries assessments alongside patient-reported outcome measures, include larger sample sizes, and adjust for both age and BMI simultaneously in the statistical analyses, in order to better account for the potential confounding effects of these variables on the association between PCOS and xerostomia.

Furthermore, it should be clearly acknowledged that modelling group status as the outcome variable rather than xerostomia severity or OHRQoL directly represents an important limitation of the present study. Although this approach was consistent with the primary aim of identifying factors associated with PCOS group membership, it does not allow for a direct quantification of the relationship between xerostomia severity and OHRQoL outcomes. Consequently, the clinical magnitude of these associations cannot be determined from the present analyses. Future studies are therefore encouraged to employ linear or ordinal regression with xerostomia severity or OHRQoL scores as outcome variables, which would yield more clinically interpretable and actionable findings. Although significant associations were observed between xerostomia severity and OHRQoL indicators, these findings should be interpreted with caution. Due to the cross-sectional design, only associations can be identified and causal relationships cannot be established. It should also be noted that the direction of the observed association between xerostomia and OHRQoL cannot be determined from the present data it is plausible that xerostomia contributes to impaired OHRQoL, however the reverse pathway is also conceivable. Longitudinal studies are therefore needed to disentangle these potential pathways and further clarify the nature of these relationships.

Overall, the results obtained in this study suggest that PCOS may be associated with increased xerostomia severity beyond the effect of age. Future studies including more comprehensive oral assessments would help to further clarify this relationship. As the study population was a narrow cross-section, expanded studies with different populations would increase the generalisability of the results. Taken together, future studies combining detailed periodontal and dental clinical assessments with multivariable regression models that use xerostomia severity and OHRQoL scores as dependent variables would provide more direct and clinically interpretable evidence regarding this relationship.

## Conclusion

Within the limitations described, the findings of the present study suggest that OHRQoL is impaired in females with PCOS, and that this impairment is associated with xerostomia. Therefore, healthcare professionals should be aware of this potential association and encourage females diagnosed with PCOS to preserve appropriate oral hygiene. Referral to dentists may contribute to improving OHRQoL values, although further longitudinal studies are needed to establish the directionality of these relationships.

## Data Availability

The datasets generated and analyzed during the current study are not publicly available due to privacy and ethical restrictions but are available from the corresponding author on reasonable request.

## Abbreviations

PCOS: Polycystic Ovary Syndrome
OHIP-14: Oral Health Impact Profile
XI: Xerostomia Inventory
OHRQoL: Oral Health-related Quality of Life
BMI: Body Mass Index
SF-36: Short Form-36 Health Survey
PCOSQ: Polycystic Ovary Syndrome Health-Related Quality of Life Questionnaire
